# A spatiotemporal analysis of administrative emergency hospitalization data (2012–2021) to assess outreach service adequacy to elderly residential care homes in Hong Kong

**DOI:** 10.1186/s12913-026-14300-z

**Published:** 2026-03-07

**Authors:** Hong-jie Yu, Jean Woo, Eric T. C. Lai, Grace L. H. Wong

**Affiliations:** 1 Institute of Health Equity, The Chinese University of Hong Kong, Hong Kong SAR, China; 2 Jockey Club Institute of Ageing, The Chinese University of Hong Kong, Hong Kong SAR, China; 3 Department of Medicine & Therapeutics, Faculty of Medicine, The Chinese University of Hong Kong, Hong Kong SAR, China

**Keywords:** Residential care homes for the elderly, Geriatric outreach teams, Emergency admissions, Spatiotemporal analysis, Hotspot analysis

## Abstract

**Background:**

Hong Kong’s rapidly aging population has heightened demand for hospital services, particularly among residents of residential care homes for the elderly (RCHEs). This study examined spatiotemporal trends and facility-level factors associated with emergency hospitalization transfers from RCHEs to inform the allocation and service planning of community geriatric assessment team (CGAT) outreach support from hospitals.

**Methods:**

A retrospective spatiotemporal cohort analysis was conducted using data from 801 RCHEs and 18 public emergency hospitals in Hong Kong from 2012 to 2021, encompassing 838,779 emergency admission episodes among adults aged 65 years and older. Admissions trends were analyzed, and spatial clustering of beds-adjusted admission rates was assessed using global Moran’s *I* and local Getis-Ord Gi* statistics. Facility-level predictors of hot/cold spot classification, including bed capacity, staffing levels, government subsidy status, proximity to hospitals, and CGAT service utilization, were evaluated using generalized linear mixed-effects models (GLMMs).

**Results:**

RCHE residents accounted for 23.6% of all emergency admissions among older adults. In the pre-pandemic period from 2012 to 2019, overall elderly admissions and hospital stays increased by 23.5% and 34.6%, respectively. Despite a sharp decline in 2020, both metrics returned to 2017 levels by 2021, with RCHE-sourced transfers consistently exceeding 20%. Significant spatial clustering was observed annually (Moran’s *I*: 0.055–0.130, all *p*-values < 0.001), with 10.9–20.0% of RCHEs identified as high-transfer-rate hot spots across the study years, concentrated in four urban districts. GLMMs revealed that smaller facilities (OR = 1.21, 95% CI: 1.05–1.40), those with lower staffing levels (OR = 1.28, 95% CI: 1.07–1.54), closer proximity to hospitals, and lower outreach service utilization were more likely to be high-transfer-rate hot spots. Conversely, greater distance to hospitals and higher outreach use predicted low-transfer-rate cold spots.

**Conclusions:**

RCHE residents are frequent users of hospital emergency services. Spatial disparities in emergency admissions are associated with facility-level factors like low staffing, limited outreach, and geographic proximity to hospitals. Identifying hot spots facilitates a targeted review of whether CGAT resources are adequately distributed and staffed, how efficiently they operate, and whether RCHEs in those areas have sufficient staffing and care quality.

**Supplementary Information:**

The online version contains supplementary material available at 10.1186/s12913-026-14300-z.

## Introduction

It is a widely accepted premise that aging populations impose increasing demands on hospital services [1]. Concurrently, evidence suggests that the rate of hospital bed expansion is unlikely to match projected needs [2], a key driver for developing outreach alternatives to reduce hospital admissions [3]. Hong Kong exemplifies this challenge, possessing one of the world’s highest life expectancies and a rapidly growing number of older adults [4]. However, this extension of total life expectancy has not been accompanied by a proportional extension of health span [5], resulting in a growing population of functionally dependent older adults [6–8]. Projections indicate that the proportion of hospital bed occupancy attributable to those aged 85 and older will triple from 15% in 2010 to 42% by 2066, with individuals aged 75 and over expected to account for 70% of total hospitalization days [9].

Due to cramped living conditions (median living space: 179 sq. feet per person) [10], the growing prevalence of older adults living alone or with a spouse [10], and increasing frailty [7], the need for residential care homes for the elderly (RCHEs) continues to grow. There is a wide variation in the staffing ratios, sizes, and quality of accommodations in RCHEs, and these factors affect the quality of care [11]. There is a general correlation between cost and quality [12]. Approximately 27% of RCHEs receive government subsidies [13], which contribute to improved staff-to-bed ratios and enhanced quality of care. Although these subsidized RCHEs are typically more affordable, they often face longer waiting lists [14, 15].

Despite growth in the number of licensed RCHEs [15], expanded capacity is not, in itself, sufficient to reduce hospitalization rates for frail older adults requiring residential care. A quality improvement initiative was started in mid-1995 by the Hospital Authority (HA), where the Geriatric Medicine service of each of the seven hospital clusters covering the whole territory provides outreach support to these RCHEs, with a view to reducing hospital admissions or re-admissions [16]. The outreach service consists of hospital doctors holding clinics at RCHEs, as well as the establishment of nurse-led community geriatric assessment teams (CGATs) [16]. Each CGAT is based in a regional hospital and serves RCHEs within the catchment area for that hospital. Regular multidisciplinary case conferences are held to promote collaboration among healthcare providers. RCHE staff can contact a designated CGAT nurse for assistance with various issues, such as changes in residents’ clinical conditions, arranging medication prescriptions, assessing residents upon discharge from the hospital, and creating advanced care plans. However, this support is typically available only on weekdays.

Reducing hospital bed utilization by RCHE residents through CGATs is contingent upon multiple factors: patient-level variables such as socioeconomic status, comorbidity burden, and polypharmacy [17]; as well as facility-level factors such as whether it receives government subvention (when staffing levels are generally higher), care quality improvements driven by advance care planning [18], geographic proximity to acute care facilities, and the intensity of hospital outreach services, which may be influenced by the distribution of RCHEs. Additionally, the spatial clustering of RCHEs may further lead to disparities in the need for outreach services among various hospital clusters.

As an initial step in investigating the utilization of hospital services by older adults residing in RCHEs, we analyzed the spatial-temporal trends in emergency hospitalizations over a ten-year period from 2012 to 2021. This analysis aims to gather data that may assist in aligning the provision of healthcare services with the demand of RCHE residents.

## Methods

### Data sources

This spatiotemporal analysis involves three categories of datasets: emergency admissions, hospital characteristics, and characteristics of RCHEs. The emergency admission data were extracted from the Clinical Data Analysis and Reporting System, which is managed by the HA of Hong Kong. HA is a statutory organization responsible for overseeing 43 public hospitals, including 18 accident and emergency (A&E) hospitals, as well as 49 specialist clinics and 73 general outpatient clinics across Hong Kong [19]. We obtained demographic information, time records, and principal diagnoses for all A&E records of older adults aged 65 and older from 2012 to 2021. Hospital characteristics during this period were sourced from the annual reports (https://www.ha.org.hk/visitor/ha_visitor_index.asp?Content_ID=212441&Lang=ENG) [20] of HA, which include data on location, recent bed capacity, annual emergency admissions, and annual geriatric outreach services. The characteristics of RCHEs, including location, bed capacity, government-funded beds, and staffing levels, were primarily obtained from publicly available information from the Social Welfare Department (SWD, https://www.elderlyinfo.swd.gov.hk/en, which is responsible for implementing governmental policies related to social welfare and with the development and coordination of social welfare services, including services for the elderly) and supplemented by two additional elderly care websites (https://elderlypage.com/index.php and https://www.e123.hk/). The study was conducted in accordance with the Declaration of Helsinki. The use of HA data, along with the waiver of individual patient informed consent, received ethical approval from the Joint Chinese University of Hong Kong-New Territories East Cluster Clinical Research Ethics Committee (Reference No.: 2022.157).

After excluding day patients with a length of stay of one day, we included a total of 3,553,475 eligible A&E episodes in our analysis, with 23.6% of these episodes originating from 905 RCHEs. We excluded 104 RCHEs due to missing information on location and bed capacity, which correspondingly led to the exclusion of 15,001 emergency episodes, resulting in an attrition rate of 1.8%. Ultimately, our analysis was based on data from 18 A&E hospitals and 801 RCHEs that recorded 823,778 transfers over a ten-year period (Supplementary Fig. 1).

### Data analysis process

Initially, we plotted the annual trends in A&E admissions and length of stay, both in total and by RCHE, alongside the trends in geriatric outreach services. Following this, we mapped the locations of the 18 A&E hospitals and 801 RCHEs to facilitate spatial analysis. We created an interactive map to visualize RCHE and hospital locations in Hong Kong. RCHEs appeared as proportional circles colored by bed capacity (Yellow-Orange-Red gradient), with size scaled to bed count. The map was centered at Hong Kong’s coordinates (22.32°N, 114.17°E) with popups displaying key facility information. We calculated a beds-adjusted admission rate expressed as the number of admissions per 10 registered beds of individual RCHE. The spatial patterns based on beds-adjusted admission rate were assessed using spatial autocorrelation, specifically Moran’s *I*, and hotspot analyses using the local Getis-Ord Gi* statistics.

We then extracted the spot types of RCHEs: cold, hot, and non-significant spots at the 95% confidence interval (CI) levels across the years, matching data on bed capacity, government-funded beds, staff numbers, and the distance to the nearest A&E hospital, as well as the outreach services of the closest hospital. This approach allowed us to construct panel data to investigate facility-level factors influencing low- or high-transfer-rate spatial cluster of RCHEs. Among the factors analyzed, only the outreach services of the closest hospital varied across the years.

### Statistical methods

We summarized the characteristics of A&E admissions, hospitals, and RCHEs. Categorical variables included sex, age groups (youngest old: 65–74 years; middle old: 75–84 years; oldest old: ≥ 85 years), admission year, admission season, payment sources (publicly assisted vs. non-publicly assisted), and principal diagnoses based on the 10th International Classification of Diseases (ICD-10). Continuous variables, such as age, length of stay, and admission frequency per patient, were reported as medians with first (Q1) and third (Q3) quartiles. Additionally, we presented the characteristics of hospitals and RCHEs, along with ten-year data on received and transferred A&E visits, as median [Q1, Q3] due to their skewed distribution.

#### Spatial autocorrelation

Global Moran’s *I* is a statistical index to assess spatial autocorrelation [21, 22], which measures the degree to which emergency transfer rates at one RCHE are similar to rates at neighboring RCHEs. A positive Moran’s *I* (ranging from − 1 to 1) indicate spatial clustering of similar values, while a negative value suggests dispersion. We calculated the statistics annually to evaluate territory-wide spatial dependency in transfer patterns. Statistical significance was determined using a standardized Z-score; a result with |Z| > 1.96 (*p*-value < 0.05) indicates significant spatial clustering that is unlikely due to random chance.

#### Hotspot analysis

To identify specific locations of statistically significant high or low hospitalization rates, hotspot analysis was performed using the Getis-Ord Gi* statistic [21, 22]. By identifying areas of high concentration or activity, it enables researchers and decision-makers to focus their efforts on regions that may require targeted interventions, thereby improving resource allocation and enhancing overall outcomes [21, 22]. This method identifies spatial clusters where high values (“hot spots”) or low values (“cold spots”) are concentrated beyond expected random distribution. In this study, a hot spot refers to an RCHE with a high beds-adjusted transfer rate that is also surrounded by other RCHEs with similarly high rates. Conversely, a cold spot indicates an area of consistently low transfer rates. Statistical significance was defined at the 95% CI: facilities with Gi* Z-scores > 1.96 were classified as hot spots, those with Z-scores < -1.96 as cold spots, and all others as non-significant. Detailed mathematical formulae for both Moran’s *I* and Getis-Ord Gi* statistics are provided in Supplementary Methods.

#### Generalized linear mixed-effects models

To analyze facility-level factors associated with the formation of spatial hot and cold spots, we employed generalized linear mixed-effects models (GLMMs) with binomial distributions and random intercepts by year to account for temporal dependencies. We calculated the ratios of government-funded beds and staff-to-bed ratios as predictors, using the number of government-funded beds and staff to determine bed counts. Due to the high correlation between these ratios (see Supplementary Table 1), we classified both as binary variables based on their median values to indicate facility size and staffing levels. The government-funded beds ratio was categorized as private or partially funded (funded ratio < 0.5) and public or mostly funded (funded ratio ≥ 0.5). Geographic accessibility included two continuous measures: (1) distance to the nearest A&E hospital (in kilometers, log-transformed for normality) and (2) outreach service intensity of the nearest hospital offering outreach services. These measures were standardized (z-scores) to facilitate comparison of effect sizes. We conduct separate analyses for hot spots (versus non-hot spots) and cold spots (versus non-cold spots) in both univariate and multivariate GLMMs after multiple imputations (10 datasets) to address missing data in the staff-to-bed ratio and government-funded beds ratio. This approach directly links facility-level predictors to systemic spatial patterns of elevated hospitalization demand, with the adjusted odds ratio (OR) quantifying the probability of belonging to a low-/high-transfer-rate cluster.

#### Additional analysis

Considering the seasonality in hospital admission [23] with a higher proportion of A&E admissions during winter, we also conducted season-specific analyses of spatial autoregression and hotspot analysis to assess whether spatial patterns varied by season. To assess the impact of the COVID-19 pandemic on hospitalization patterns, we conducted a comparative sub-analysis. Admissions were stratified into two periods: the pre-pandemic era (2012–2019) and the pandemic era (2020–2021). We compared the seasonal distribution of admissions and the proportional distribution of principal diagnoses (categorized by ICD-10 chapter) between these two periods using the Chi-square test.

All analyses were performed using *R* software (Version 4.4.1). The “*sf*,” “*leaflet*,” and “*spdep*” packages were specifically used for spatial analysis.

## Results

Our study included a total of 838,779 A&E admissions from RCHEs. Among these episodes, elderly individuals aged 85 years and older represented approximately 60% of total visits, with over 70% being recipients of public assistance (see Table 1). Notably, there was a higher frequency of A&E transfers during the winter months. Respiratory diseases were identified as the most prevalent diagnosis for A&E admissions, accounting for 25.8% of episodes, followed by ill-defined symptoms, signs, and clinical findings, which constituted approximately 20%. These two rates were at least twice that of other ICD-10 disease categories. The median length of stay per episode was four days, with each patient experiencing an average of three admissions during the observation period or prior to death. The analysis encompassed 905 RCHEs, of which 801 provided complete data suitable for spatial analysis (see Supplementary Fig. 1). Each RCHE recorded a median of 831 A&E transfer episodes [Q1, Q3: 419, 1,387], involving 197 unique patients per facility [Q1, Q3: 113, 329], indicating a high ratio of A&E transfer episodes to unique patients (see Table 1).

**Table 1:** Summary of the characteristics of included A&E admissions, hospitals, and RCHEs in Hong Kong from 2012 to 2021

Characteristics	*n* (%) or median [Q1, Q3]
**A&E admission episodes from RCHEs (** ***n*** ** = 838,779)**	
** Sex**	
Female	484,903 (57.8%)
Male	353,876 (42.2%)
** Age, ** ***years***	86.0 [81.0, 91.0]
Youngest old (65–74 years)	87,359 (10.4%)
Middle old (75–84 years)	253,731 (30.3%)
Oldest old (≥ 85 years)	497,689 (59.3%)
** Admission year**	
2012	86,044 (10.3%)
2013	83,237 (9.9%)
2014	84,408 (10.1%)
2015	82,577 (9.8%)
2016	82,134 (9.8%)
2017	85,659 (10.2%)
2018	85,759 (10.2%)
2019	87,688 (10.5%)
2020	76,791 (9.2%)
2021	84,482 (10.1%)
** Admission season**	
Spring	213,784 (25.5%)
Summer	204,700 (24.4%)
Fall	198,405 (23.7%)
Winter	221,890 (26.5%)
** Payment source**	
Non-publicly assisted	231,298 (27.6%)
Publicly assisted	607,481 (72.4%)
Length of stay per episode, *days*	4 [2, 7]
Admission frequency per patient	3 [2, 6]
** Principal diagnosis by ICD-10** ^‡^	
J00 - J99: Diseases of the respiratory system	216,695 (25.8%)
R00 - R99: Symptoms, signs, and clinical findings not elsewhere classified	165,414 (19.7%)
I00 - I99: Diseases of the circulatory system	82,116 (9.8%)
N00 - N99: Diseases of the genitourinary system	74,938 (8.9%)
K00 - K93: Diseases of the digestive system	59,192 (7.1%)
S00 - T98: Injury, poisoning, and certain other consequences of external causes	53,293 (6.4%)
A00 - B99: Infectious and parasitic diseases	48,043 (5.7%)
E00 - E90: Endocrine, nutritional, and metabolic diseases	35,343 (4.2%)
L00 - L99: Diseases of the skin and subcutaneous tissue	23,363 (2.8%)
M00 - M99: Diseases of the musculoskeletal system	21,314 (2.5%)
C00 - D48: Neoplasms	17,997 (2.1%)
Others	40,828 (4.9%)
**Hospital Characteristics**	
** Hospitals offering A&E services (*n* = 18)** ^*^	
Recent No. of hospital beds per hospital	1,032 [672.5, 1,773.2]
Average annual A&E episodes per hospital	123,893 [106,626, 131,824]
Ten-year received transferred episodes from included RCHEs per hospital	46,347 [27,282, 63,924]
Ten-year received transferred unique patients from included RCHEs per hospital	11,798 [8,940, 14,841]
Hospitals offering outreach services (*n* = 17) ^†^	
Average annual outreach services per hospital	42,510 [33,271, 47,829]
RCHE Characteristics (*n* = 801)	
Recent RCHEs beds per RCHE	80 [50, 121]
Government-funded beds per RCHE^‡^	14 [0, 75]
Staff number per RCHE^‡^	32 [16, 56]
Ten-year transferred episodes per RCHE	831 [419, 1387]
Ten-year transferred unique patients RCHE	197 [113, 329]

*Among the 18 hospitals offering A&E services, one commenced operation in 2013 and another in 2016. Their statistical data were collected from 2014 to 2021 and from 2017 to 2021, respectively

†Outreach services encompass both geriatric outreach assessment services and visits from a visiting medical officer. Seventeen hospitals provide outreach services, comprising 13 A&E hospitals and 4 non-emergency hospitals. Notably, one of the A&E hospitals commenced operations in 2013

^‡^Contain missing value: 243 (< 0.1%) for the principal diagnosis, 4 (0.5%) for the government-subsided beds and 112 (14.0%) for the staff number

A&E: accident and emergency; RCHEs: residential care homes for the elderly

**Fig. 1:**
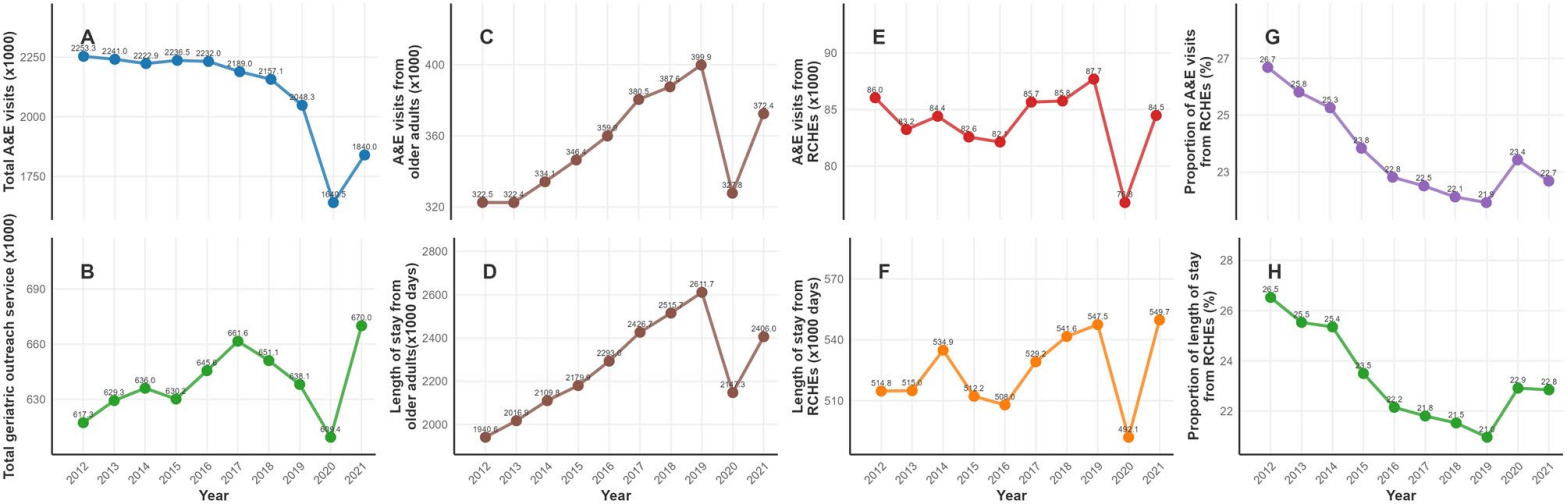
Trends in A&E visits and outreach service from 2012 to 2021. **A**: Total A&E visits; **C**: A&E visits from older adults; **E**: A&E visits from older adults in RCHEs; **G**: Proportion of A&E visits from RCHEs. **B**: Outreach services; **D**: Length of stay from older adults; **F**: Length of stay from older adults in RCHEs; **H**: Proportion of length of stay from RCHEs. A&E: accident and emergency; RCHEs: residential care homes for the elderly

Trend analysis revealed a decline in the overall number of A&E admissions; however, admissions among total older adults increased significantly by 23.5%, rising from approximately 322,000 in 2012 to 400,000 in 2019, before being disrupted by the COVID-19 outbreak (Fig. 1). Similarly, the total length of stay rose significantly by 34.6%, increasing from approximately 1,941,000 days to 2,612,000 days during the same period. The COVID-19 pandemic was associated with a disruption in the seasonal pattern of hospitalizations, attenuating the typical winter-spring peak, and with a significant shift in diagnostic profiles, most notably a decrease in the proportion of admissions for respiratory diseases from 26.8% to 21.7% (Supplementary Table 2). Despite a decrease in the proportion of A&E transfers from RCHEs, these transfers continued to represent over 20% of total A&E visits and total length of stay by older adults.

**Fig. 2:**
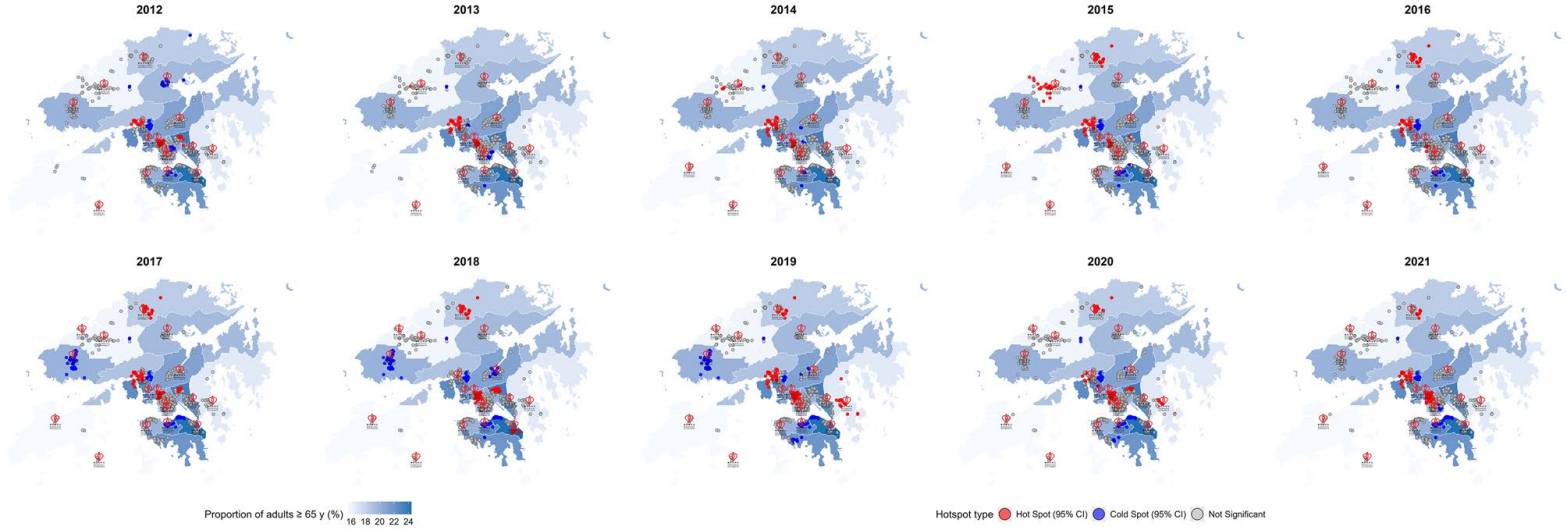
Hotspot analysis* map of A&E transfer rate from RCHEs to hospitals in Hong Kong older adults by years. *Spot types were derived from hotspot analyses to assess spatial clustering based on annual A&E transfer rate from RCHEs to hospitals. A&E: accident and emergency; RCHEs: residential care homes for the elderly

The spatial distribution of 18 A&E hospitals and 801 RCHEs is illustrated in Supplementary Fig. 2. Spatial analysis indicated significant annual clustering of A&E admissions rate, as evidenced by Moran’s *I* values ranging from 0.055 to 0.130 (all *p*-values < 0.001; Table 2). Hot spots, characterized as high-transfer-rate clusters, constituted between 10.9% and 20.0% of RCHEs annually, with a slight increase observed before 2019. These areas consistently exhibited high annual transfer rates (median: 12.2–15.1 admissions per 10 beds), in clear contrast to the much lower rates in cold spots (median: 7.6–9.8 admissions per 10 beds). The concentration of hot spots was notably persistent in the Tsuen Wan and Sham Shui Po areas. Following 2015, the Sheung Shui area also exhibited significant hot spot activity. In addition, certain years saw the emergence of hot spots in Wong Tai Sin and Tseung Kwan O. Conversely, the prevalence of hot spots in the Yuen Long area diminished between 2012 and 2015, which may be attributed to the establishment of new hospitals in the region (Fig. 2). Furthermore, spatial autocorrelation exhibited consistency across various seasons, with Moran’s *I* values ranging from 0.096 to 0.104, all of which were statistically significant (*p*-values < 0.001, as detailed in Supplementary Table 3). Notably, the distribution of hot spots remained relatively similar across seasons and was concentrated in comparable urban areas (see Supplementary Fig. 3).

**Table 2:** Spatial autocorrelation and hotspot analysis of A&E transfer rate from RCHEs to hospitals in Hong Kong older adults from 2012 to 2021

Year	No. of RCHEs	No. of hospitals	Global Moran’s I index			Annual transfer rate^#^	Hot spots^*^		Cold spots^*^		Non-significant spots^*^	
			Moran’s I	z-score	*p*-value		No. (%)	Annual transfer rate^#^	No. (%)	Annual transfer rate^#^	No. (%)	Annual transfer rate^#^
2012	700	16	0.072	8.388	< 0.001	11.8 [8.9, 15.3]	76 (10.9%)	15.1 [10.8, 17.6]	97 (13.9%)	9.3 [7.6, 12.3]	527 (75.3%)	11.9 [9.3, 15.3]
2013	712	16	0.055	6.484	< 0.001	11.7 [8.8, 14.7]	78 (11.0%)	13.8 [11.2, 16.8]	38 (5.3%)	9.8 [6.9, 12.6]	596 (83.7%)	11.5 [8.7, 14.5]
2014	707	17	0.071	8.352	< 0.001	12.0 [9.3, 15.1]	83 (11.7%)	14.0 [12.1, 17.4]	16 (2.3%)	7.6 [6.0, 9.7]	608 (86.0%)	11.7 [9.1, 14.8]
2015	717	17	0.088	10.238	< 0.001	11.8 [8.6, 14.6]	143 (19.9%)	13.1 [10.6, 16.7]	67 (9.3%)	8.8 [6.2, 11.9]	507 (70.7%)	11.7 [8.5, 14.5]
2016	723	17	0.077	8.994	< 0.001	11.5 [8.8, 14.4]	94 (13.0%)	12.9 [10.9, 15.7]	62 (8.6%)	8.5 [5.7, 11.5]	567 (78.4%)	11.5 [8.9, 14.5]
2017	732	18	0.103	12.161	< 0.001	12.0 [9.0, 14.9]	123 (16.8%)	13.7 [10.6, 17.3]	129 (17.6%)	9.5 [6.7, 11.9]	480 (65.6%)	12.3 [9.4, 15.0]
2018	736	18	0.129	15.272	< 0.001	12.1 [9.1, 14.9]	145 (19.7%)	14.3 [11.7, 17.1]	155 (21.1%)	9.6 [6.4, 12.0]	436 (59.2%)	12.3 [9.5, 14.7]
2019	740	18	0.13	15.301	< 0.001	12.2 [9.3, 15.1]	148 (20.0%)	14.7 [11.7, 18.2]	147 (19.9%)	9.6 [6.6, 12.3]	445 (60.1%)	12.3 [9.5, 15.0]
2020	760	18	0.072	8.388	< 0.001	10.5 [7.8, 13.2]	127 (16.7%)	12.2 [9.2, 15.6]	106 (13.9%)	8.3 [5.8, 10.3]	527 (69.3%)	10.8 [8.1, 13.3]
2021	764	18	0.055	6.484	< 0.001	11.3 [8.6, 14.0]	132 (17.3%)	13.2 [11.2, 16.5]	112 (14.7%)	9.1 [6.3, 11.2]	520 (68.1%)	11.2 [8.7, 13.9]

*Spot types were derived from hotspot analyses to assess spatial clustering based on annual A&E transfer rate from RCHEs to hospitals

#The annual A&E transfer rate was presented as median [interquartile range], which adjusted for bed capacity and is calculated as the number of admissions per 10 registered beds in each RCHE

A&E: accident and emergency; RCHEs: residential care homes for the elderly

GLMMs models revealed that distinct facility-level predictors for being a high or low transfer-rate cluster. Smaller RCHEs (< 80 beds) and those with lower staffing ratios demonstrated significantly higher odds of being hot spots (OR = 1.21, 95% CI: 1.05–1.40; OR = 1.28, 95% CI: 1.07–1.54, respectively). A strong geographical pattern emerged: proximity to a hospital was associated with being hot spots, while greater distance was a key predictor for being cold spots. Similarly, lower utilization of hospital outreach services was strongly associated with hot spots, whereas higher utilization was linked to cold spots (see Supplementary Tables 4 and Table 3).

**Table 3:** Univariate and multivariate analyses of the association between characteristics and A&E transfer rate of RCHEs

RCHE characteristics	Cold spots*				Hot spots*			
	Univariate†		Multivariate†		Univariate†		Multivariate†	
	OR (95% CI)	*p*-value	OR (95% CI)	*p*-value	OR (95% CI)	*p*-value	OR (95% CI)	*p*-value
Beds								
Large scale with beds ≥ 80	Ref		Ref		Ref		Ref	
Small scale with beds < 80	1.05 (0.91, 1.21)	0.481	0.84 (0.72, 0.99)	0.033	1.09 (0.96, 1.23)	0.194	1.21 (1.05, 1.40)	0.008
Government-funded beds ratio								
Public or mostly funded with ratio ≥ 0.5	Ref		Ref		Ref		Ref	
Private or partially funded with ratio < 0.5	1.15 (0.99, 1.33)	0.073	1.06 (0.87, 1.28)	0.546	1.08 (0.94, 1.23)	0.270	0.83 (0.69, 1.00)	0.052
Staff-beds ratio								
High staffing level with ratio ≥ 0.4	Ref		Ref					
Low staffing level with ratio < 0.4	1.27 (1.09, 1.47)	0.002	1.17 (0.98, 1.40)	0.088	1.16 (1.01, 1.34)	0.036	1.28 (1.07, 1.54)	0.007
Distance to the nearest A&E hospital z-score‡	1.19 (1.12, 1.26)	< 0.001	1.09 (1.02, 1.17)	0.014	0.51 (0.47, 0.56)	< 0.001	0.51 (0.46, 0.56)	< 0.001
Outreach service of nearest hospital z-score‡	2.06 (1.94, 2.20)	< 0.001	2.04 (1.91, 2.19)	< 0.001	0.58 (0.53, 0.63)	< 0.001	0.53 (0.48, 0.58)	< 0.001

Note:

*Spot types were derived from hotspot analyses to assess spatial clustering based on annual A&E transfer rate from RCHEs to hospitals

†The association was evaluated using generalized linear mixed-effects models, incorporating random effects based on years, following ten imputations for missing data regarding staff numbers and government funding ratios

‡The distance to the nearest hospitals was computed using straight-line measurements. The outreach services provided by the nearest hospitals were utilized as a proxy for the outreach service levels in RCHEs

A&E: accident and emergency; RCHEs: residential care homes for the elderly; CI: confidence interval; OR: odds ratio; Ref: reference group

## Discussion

This study demonstrates that a distinct subset of RCHEs in Hong Kong are high-frequency users of hospital services. From 2012 to 2021, between 10.9% and 20.0% of all RCHEs were consistently identified as high-transfer-rate “hot spots,” a proportion that followed a volatile but pronounced upward trajectory, nearly doubling by 2019. These hot spots were persistently concentrated in several urban districts, indicating a geographically stable yet intensifying demand on acute care services.

Concurrently, public hospitals are experiencing increasing overcrowding, characterized by the utilization of extra beds in wards and corridors alongside pressure for timely discharge after acute illness resolution [24]. From an administrative perspective, there is an urgent need to investigate whether emergency transfers from RCHEs can be reduced. Our data underscore the necessity of reviewing the existing support arrangements provided to RCHEs by the CGAT from public hospitals. Although the need for such support was acknowledged decades ago with the establishment of CGATs and on-site clinics, there has yet to be an overall audit of the need and supply of CGATs services, taking into account the characteristics of RCHEs, their locations, and the utilization of hospital outreach services. The strong inverse correlation between outreach service utilization and emergency transfers suggests the potential impact of these service.

The COVID-19 pandemic offered critical insights into achievable outcomes. During 2020–2021, visits to A&E departments fell sharply. Our comparative analysis revealed this decline was accompanied by a significant shift in diagnostic patterns: hospitalizations for respiratory diseases decreased markedly (from 26.8% to 21.7% of admissions), likely reflecting increases in on-site facility care and the efficacy of non-pharmacological interventions such as universal masking and enhanced hygiene in suppressing seasonal pathogens [25]. However, this period also saw a concurrent rise in admissions for ill-defined symptoms/signs and genitourinary diseases, suggesting that stringent infection control measures may have inadvertently strained the capacity of RCHES for managing other complex, non-infectious conditions within RCHEs. This period demonstrated that while virtual support can partially sustain outreach, the pandemic’s broader disruption highlighted vulnerabilities in the comprehensive, in-situ management of frail older adults.

The seasonal spike in RCHE admissions is closely tied to respiratory infections [26, 27] and is also influenced by meteorological parameters like temperature, air pollution, and humidity [27, 28]. Nonetheless, the fundamental vulnerability of frail older adults to environmental factors persists [29]. As adequate heating in RCHEs is not common practice, future measures to improve thermal comfort may help mitigate a portion of excess winter admissions [30]. This environmental intervention, combined with sustained infection control and robust clinical outreach, represents a multifaceted strategy for reducing seasonal hospitalization pressure.

Our findings reveal significant spatial variation in the number and characteristics of RCHEs supported by individual public hospitals. The concentration of high-transfer-rate “hot spots” in specific urban districts results in clusters of elevated medical resource utilization, increasing regional medical pressure and underscoring the need for enhanced outreach services. These hot spots are characterized by being smaller, having lower staff-to-bed ratios, and being located closer to hospitals, yet they utilize fewer outreach services. This suggests that proximity alone does not guarantee adequate support; these facilities may be unable to leverage their geographic advantage, possibly due to resource constraints (e.g., staff shortages limiting the capacity to coordinate outreach visits). Conversely, “cold spots” are farther from hospitals and are associated with higher utilization of outreach services, suggesting that proactive outreach can effectively reduce emergency transfer rates even when geographic access is challenging. This pattern indicates a potential mismatch between the supply and demand for current CGAT services.

From the hospital perspective, a comprehensive review of management strategies for the two most prevalent admission diagnoses is warranted. Current disease-based guidelines often fail to account for the complexities of older adult care [31]. For example, the ICD code for respiratory diseases includes conditions like aspiration pneumonia, which is frequent in the RCHEs population, particularly among patients at the end of life [32]. Management strategies should incorporate not only antibiotic protocols but also a thorough review of advanced care planning, swallowing assessments, and vaccination requirements [33]. Additionally, structured pathways for managing geriatric syndromes (commonly coded R00-99) are lacking unless patients are under geriatricians conducting comprehensive assessments. Consequently, disease-specific management often takes precedence over the management of geriatric syndromes.

Government policies play a crucial role, as hospitals and RCHEs are overseen by different departments with distinct budgets: the HA and the SWD. The Department of Health (DH) is responsible for infection control policies [34]; however, these policies may inadvertently increase hospital admissions if RCHEs cannot implement them due to resource constraints, making hospital transfer a default solution. Furthermore, until the end of 2025, an outdated legal requirement mandated that all deaths in RCHEs be reported to the police, often prompting transfers of residents nearing end-of-life. An amendment at the end of 2025, allowing physicians who have cared for a resident within the preceding 14 days to provide death certification, could reduce such transfers, facilitating more dignified deaths within RCHEs. This process could be further supported by advance care planning. Given that HA, SWD, and DH all provide outreach services to RCHEs, improved inter-departmental coordination could minimize duplication and enhance efficiency. Resource allocation for individual hospitals could also be adjusted based on the specific demands of RCHEs in their catchment areas.

From the perspective of RCHEs, the development of teleconsultation services offered by various providers in both the public and private sectors may significantly reduce the reliance on hospital services [35], as evidenced during the COVID-19 pandemic.

### Strength and limitations

This study utilizes a comprehensive, territory-wide administrative dataset encompassing all public hospital emergency admissions over a decade, providing robust population-level evidence. The application of spatiotemporal analysis identifies specific geographic clusters of high utilization, offering actionable insights for resource allocation. By integrating hospitalization records with facility-level characteristics, the analysis shifts the focus toward modifiable structural and operational factors within the residential care environment.

This study also has several limitations. First, the ecological and descriptive study design, while identifying important spatial patterns and associations, does not permit causal inference regarding the effectiveness of specific interventions. We did not examine in detail the heterogeneity of outreach service models, hospital-level policies, or broader governmental funding mechanisms that may influence hospitalization rates. Second, data constraints imposed methodological limitations. Crucial patient-level clinical factors [17], such as frailty scores, comorbidity profiles, and the presence of advanced care plans, were unavailable and may explain residual variation between facilities. Furthermore, facility characteristics like staffing ratios were derived from a recent administrative snapshot rather than longitudinal tracking, and registered bed capacity served as a denominator in place of actual occupancy, potentially obscuring dynamic changes in resident acuity and dependency. Third, our geographic measures may not fully capture operational realities. Proximity was calculated using Euclidean distance, which does not account for travel time, terrain, or transportation networks. The analysis also assumed a primary linkage between each RCHE and its nearest hospital, whereas in practice, facilities may utilize multiple hospitals for transfers or outreach services based on established relationships or patient preference. Finally, the findings are intrinsically linked to the unique context of Hong Kong’s densely populated urban environment and its specific healthcare and social welfare structures. The generalizability of these results to other regions with differing models of long-term care, hospital funding, and geographic dispersion may be limited.

## Conclusion

In conclusion, RCHEs residents are significant contributors to hospital emergency admissions, which display distinct spatiotemporal patterns and are closely linked to facility-level characteristics. Smaller, under-staffed homes situated near hospitals but utilizing fewer outreach services are at a greater risk of becoming high-transfer-rate hot spots. Identifying these hot spots establishes an evidence-based foundation for targeted resource optimization. This optimization involves a dual-focused review: first, an assessment of the geographic distribution, staffing adequacy, and operational efficiency of CGAT resources to ensure they effectively meet localized needs; and second, an evaluation of the RCHEs within high-risk catchments, focusing on their staffing levels and adherence to quality-of-care standards.

## Supplementary Information

Below is the link to the electronic supplementary material.

Supplementary Material 1

Supplementary Material 2

Supplementary Material 3

Supplementary Material 4

## Data Availability

The datasets for the current study are available from the corresponding author upon reasonable request, contingent upon receiving approval from the Hong Kong Hospital Authority.

## Abbreviations

A&E: Accident and Emergency
CGATs: Community Geriatric Assessment Teams
CI: Confidence Interval
DH: Department of Health (Hong Kong)
GLMMs: Generalized Linear Mixed-effects Models
HA: Hospital Authority (Hong Kong)
ICD-10: International Classification of Diseases, 10th Revision
OR: Odds Ratio
RCHE: Residential Care Home for the Elderly
SWD: Social Welfare Department (Hong Kong)
