# Supplementary figures and images for: A spatiotemporal analysis of administrative emergency hospitalization data (2012–2021) to assess outreach service adequacy to elderly residential care homes in Hong Kong
Source: BMC Health Serv Res. 2026 Mar 7;26:521. doi: 10.1186/s12913-026-14300-z (PMC13081407; doi:10.1186/s12913-026-14300-z)

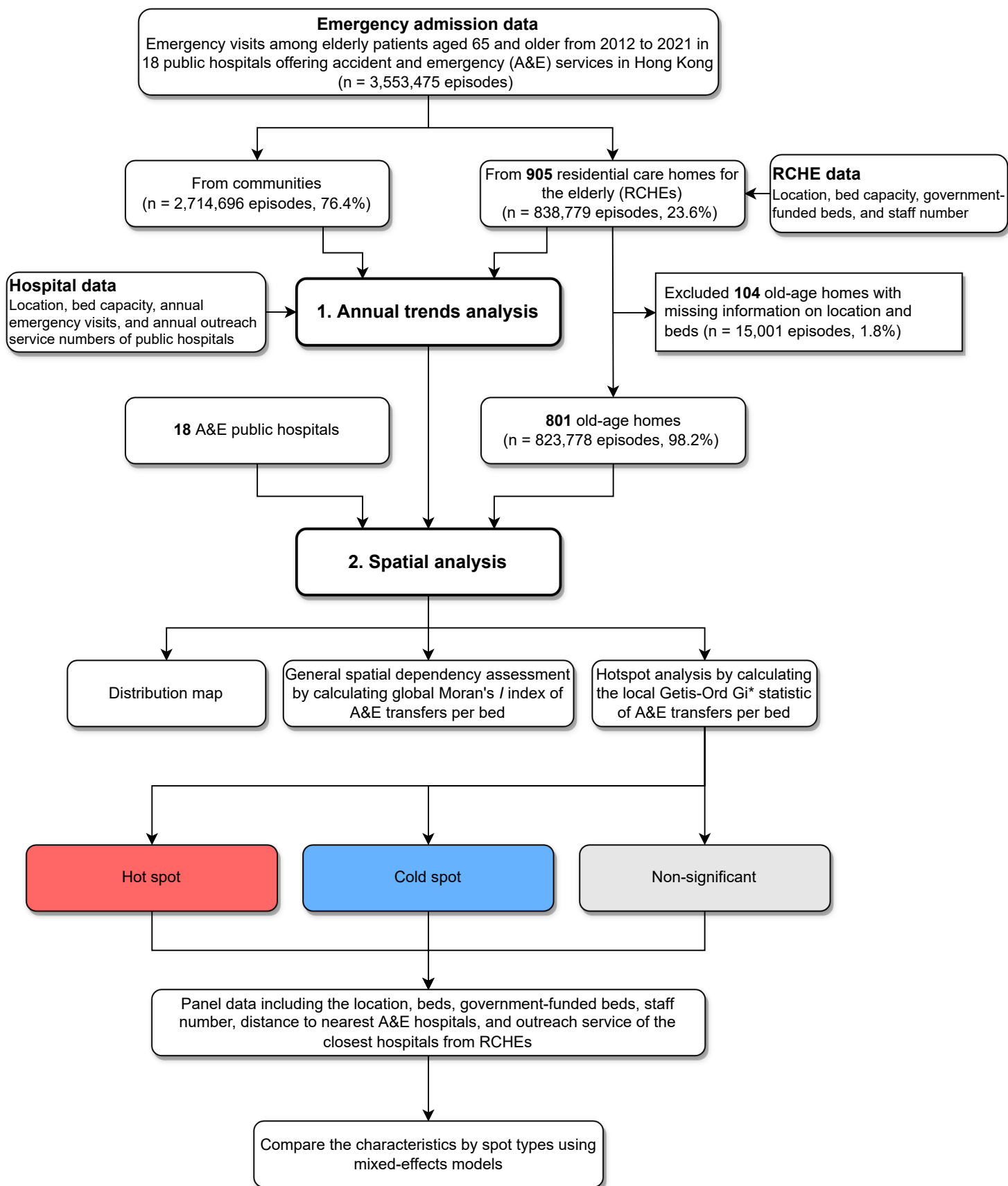

Supplement: Supplementary file 1 — Supplementary Material 1 [file 12913_2026_14300_MOESM1_ESM.pdf]
