# Supplementary material for: A spatiotemporal analysis of administrative emergency hospitalization data (2012–2021) to assess outreach service adequacy to elderly residential care homes in Hong Kong
Source: BMC Health Serv Res. 2026 Mar 7;26:521. doi: 10.1186/s12913-026-14300-z (PMC13081407; doi:10.1186/s12913-026-14300-z)
Supplement: Supplementary file 4 — Supplementary Material 4 [file 12913_2026_14300_MOESM4_ESM.docx]

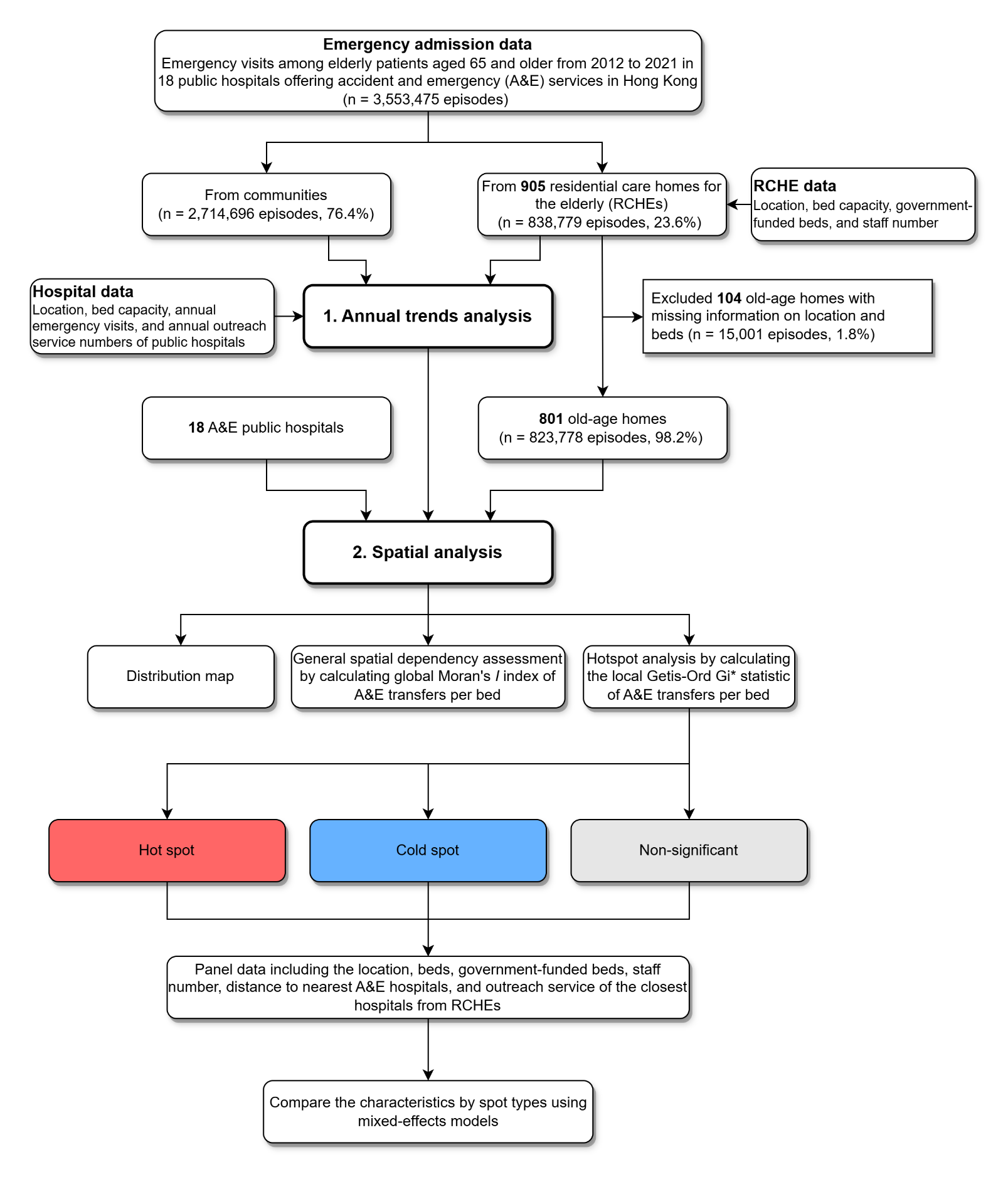
**Supplementary Materials**

**Supplementary Figure 1**. Data processing and analysis flowchart

**Supplementary Methods** for the spatial analysis

We first used Moran's *I* statistics to quantify territory-wide spatial dependency in annual patterns of Accident & Emergency (A&E) transfers among residential care homes for the elderly (RCHEs). The formal expression is given by:

$\begin{aligned} I=\frac{n}{S_{0}}\frac{\sum_{i=1}^{n} \sum_{j=1}^{n} w_{ij}\left( x\_i-\bar{x} \right)\left( x_{j}-\bar{x} \right)}{\sum_{i=1}^{n} \left( x_{i}-\bar{x} \right)^{2}}\#\left( 1 \right) \end{aligned}$where $I$ represents the Moran’s *I* statistic (ranging from -1 to 1), $n$ denotes the number of RCHEs, $w_{ij}$ is the spatial weight between RCHE $i$ and $j$, $x_{i}$ indicates A&E transferred episodes count at location$i$. $\bar{x}$ signifies the mean value of A&E transferred episodes count across all locations, and $S_{0}$equals the sum of all spatial weight with the formula as:

$$\begin{aligned} S_{0}=\sum_{i} \sum_{j} w_{ij}\#\left( 2 \right) \end{aligned}$$

A standardized Z-score was computed to test the significance via:

$$\begin{aligned} Z_{I}=\frac{I-E\left[ I \right]}{\sqrt{\text{Var}\left( I \right)}}\#\left( 3 \right) \end{aligned}$$

Where $E\left[ I \right]=-1/\left( n-1 \right)$ is the expected value under spatial randomness. Results with $\left| Z_{I} \right| > 1.96$ (*p*-value < 0.05) indicate statistically significant clustering.

We then identified statistically significant spatial clusters of high A&E transfer rate (hot spots) and low transfer rate (cold spots) at individual RCHE via the calculation of Getis-Ord Gi* statistics as:

$$\begin{aligned} G_{i}^{*}=\frac{\sum_{j=1}^{n} w_{ij}\left( x_{j}-\bar{x} \right)}{s\sqrt{\frac{n\sum_{j=1}^{n} w_{ij}^{2}-\left( \sum_{j=1}^{n} w_{ij} \right)^{2}}{n-1}}}\#\left( 4 \right) \end{aligned}$$

Where $G_{i}^{*}$ denotes the Gi* statistic for RCHE $i$, $s$ is the standard deviation of A&E transferred episodes, and other terms maintain consistent with Moran’s *I* equation (1).

Statistical significance was determined by using standardized Gi* Z-scores at a 95% CI level:

$G_{i}^{*}$ > 1.96 with *p*-value <0.05 classified as hot spots;

$G_{i}^{*}$ < -1.96 with *p*-value <0.05 classified as cold spots;

All other RCHEs were deemed non-significant spots.

**Supplementary Table 1**. The correlation matrix for RCHE characterises

| **RCHE Characteristics** | **Beds** | **Government-funded beds ratio** | **Staff-beds ratio** | **Distance to the nearest A&E hospital, m** | **Outreach service of nearest hospital (x100)** |
| --- | --- | --- | --- | --- | --- |
| **Beds** | 1 |  |  |  |  |
| **Government-funded beds ratio** | 0.398 | 1 |  |  |  |
| **Staff-beds ratio** | 0.027 | 0.617 | 1 |  |  |
| **Distance to the nearest A&E hospital, m** | -0.003 | 0.039 | -0.025 | 1 |  |
| **Outreach service of nearest hospital (x100)** | -0.114 | -0.066 | -0.061 | 0.051 | 1 |

A&E: accident and emergency; RCHEs: residential care homes for the elderly.

**Supplementary Table 2**. The seasonal and diagnostic profile of A&E transfers among RCHE residents: pre-pandemic vs. pandemic periods

|  | **Pre-COVID-19^*^**  **(n = 677506)** | **COVID-19^*^**  **(n = 161273)** | **Chi-square test** |
| --- | --- | --- | --- |
| **Admission season** |  |  |  |
| Spring | 174467 (25.8%) | 39317 (24.4%) | X-squared = 391.05 *p*-value <0.001 |
| Summer | 165514 (24.4%) | 39186 (24.3%) |  |
| Fall | 157377 (23.2%) | 41028 (25.4%) |  |
| Winter | 180148 (26.6%) | 41742 (25.9%) |  |
| **ICD-10 diagnosis^#^** |  |  |  |
| A00 - B99: Infectious and parasitic diseases | 38834 (5.7%) | 9209 (5.7%) | X-squared = 2846.1 *p*-value <0.001 |
| C00 - D48: Neoplasms | 15007 (2.2%) | 2990 (1.9%) |  |
| E00 - E90: Endocrine, nutritional, and metabolic diseases | 27621 (4.1%) | 7722 (4.8%) |  |
| I00 - I99: Diseases of the circulatory system | 67635 (10.0%) | 14481 (9.0%) |  |
| J00 - J99: Diseases of the respiratory system | 181684 (26.8%) | 35011 (21.7%) |  |
| K00 - K93: Diseases of the digestive system | 47634 (7.0%) | 11558 (7.2%) |  |
| L00 - L99: Diseases of the skin and subcutaneous tissue | 18643 (2.8%) | 4720 (2.9%) |  |
| M00 - M99: Diseases of the musculoskeletal system | 17072 (2.5%) | 4242 (2.6%) |  |
| N00 - N99: Diseases of the genitourinary system | 58759 (8.7%) | 16179 (10.0%) |  |
| R00 - R99: Symptoms, signs, and clinical findings not elsewhere classified | 129012 (19.0%) | 36402 (22.6%) |  |
| S00 - T98: Injury, poisoning, and certain other consequences of external causes | 42439 (6.3%) | 10854 (6.7%) |  |
| Others | 32992 (4.9%) | 7836 (4.9%) |  |

Data are presented as n (%). The analysis includes all A&E episodes with a length of stay >1 day.

*Pre-pandemic period: 2012–2019. Pandemic period: 2020–2021.

#Principal diagnosis is categorized by chapter of the International Classification of Diseases, 10th Revision (ICD-10).

A&E: accident and emergency; RCHEs: residential care homes for the elderly.


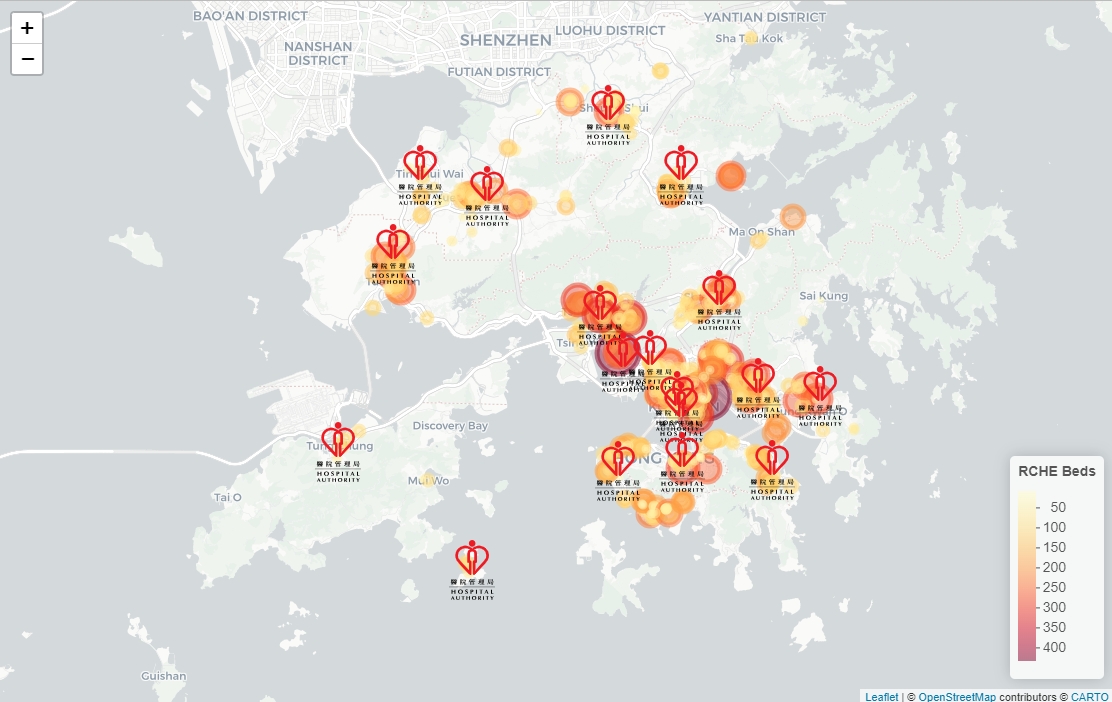
**Supplementary Figure 2**. Distribution of 801 RCHEs^*^ and 18 A&E hospitals

*RCHEs appeared as proportional circles colored by bed capacity (Yellow-Orange-Red gradient), with size scaled to bed count.

The map was centered at Hong Kong's coordinates (22.32°N, 114.17°E) with popups displaying key facility information.

A&E: accident and emergency; RCHEs: residential care homes for the elderly.

**Supplementary Table 3**. Spatial autocorrelation analysis of ten-year A&E transfers rate from RCHEs to hospitals in Hong Kong older adults by seasons

| **Season** | **No. of RCHEs** | **No. of hospitals** | **Global Moran's *I* index** | | |  | **Hotspot analysis**^*^ | | | |
| --- | --- | --- | --- | --- | --- | --- | --- | --- | --- | --- |
|  |  |  | **Moran’s *I*** | **z-score** | ***p*-value** |  | **Median [Q1, Q3] of ten-year A&E transfer rate per RCHE**^#^ | **Hot spots (%)** | **Cold spots (%)** | **Non-significant spots (%)** |
| Spring | 801 | 18 | 0.096 | 11.848 | <0.001 |  | 28.8 [20.5, 35.3] | 137 (17.1%) | 99 (12.4%) | 565 (70.5%) |
| Summer | 801 | 18 | 0.101 | 12.502 | <0.002 |  | 26.9 [19.0, 33.6] | 147 (18.4%) | 102 (12.7%) | 552 (68.9%) |
| Fall | 801 | 18 | 0.104 | 12.857 | <0.003 |  | 25.9 [19.3, 32.4] | 138 (17.2%) | 109 (13.6%) | 554 (69.2%) |
| Winter | 801 | 18 | 0.096 | 11.903 | <0.004 |  | 29.3 [21.2, 36.5] | 137 (17.1%) | 100 (12.5%) | 564 (70.4%) |

*Spot types were derived from hotspot analyses to assess spatial clustering based on ten-year A&E transfer rate from RCHEs to hospitals.

#The ten-year A&E transfer rate was adjusted for bed capacity and is expressed as the number of admissions per 10 registered beds in each RCHE.

A&E: accident and emergency; RCHEs: residential care homes for the elderly.


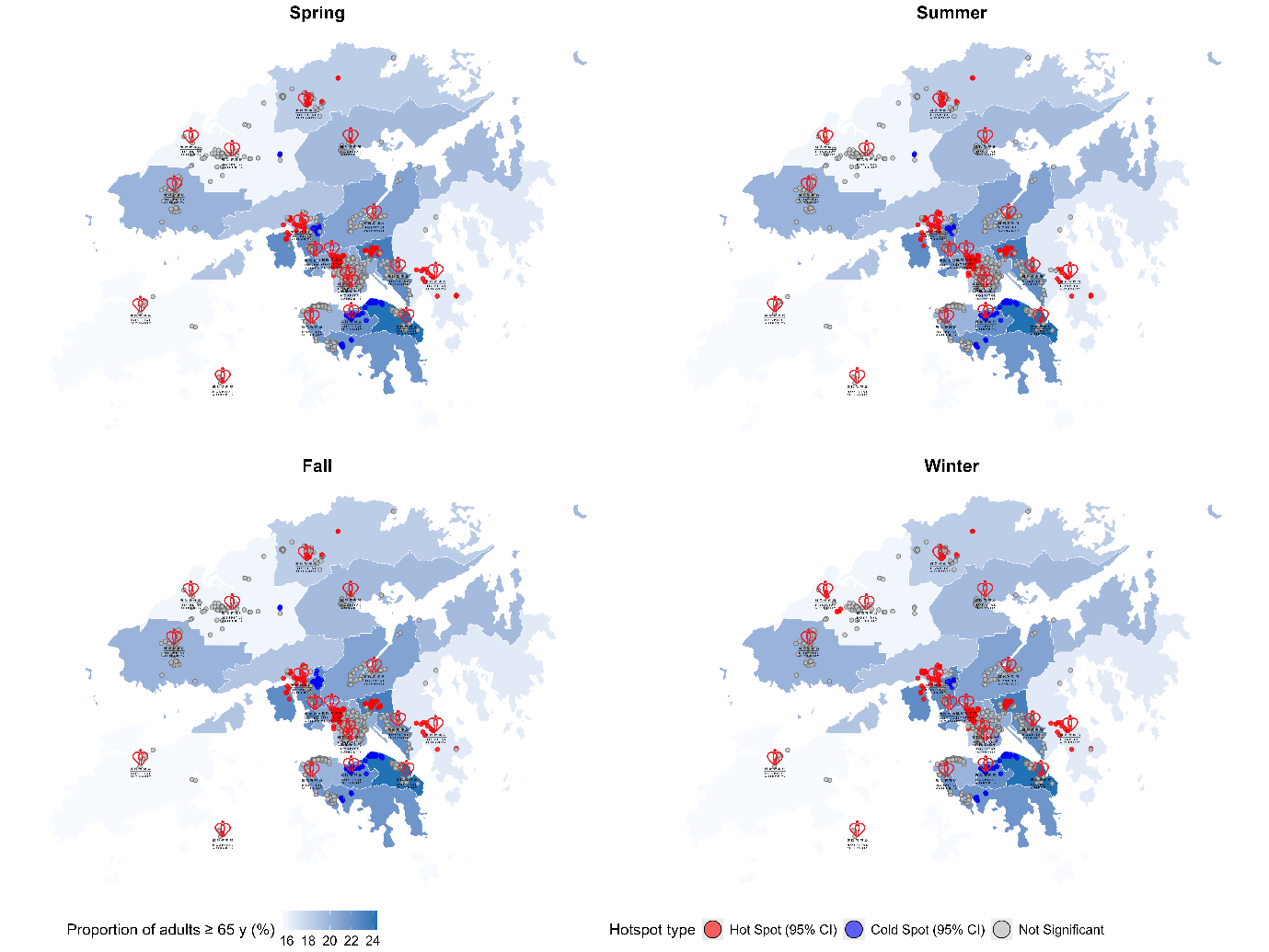
**Supplementary Figure 3**. Hotspot analysis^*^ of ten-year A&E transfer rate from RCHEs to hospitasl in Hong Kong older adults by seasons

*****Spot types were derived from hotspot analyses to assess spatial clustering based on ten-year A&E transfer rate from RCHEs to hospitals.

A&E: accident and emergency; RCHEs: residential care homes for the elderly.

**Supplementary Table 4**. Characteristics of RCHEs by spot types^#^ from 2011 to 2021

| **RCHEs characteristics** | **Cold spots (n = 788)** | **Hot spots (n = 870)** | **Non-significant spots (n = 5,633)** | **Overall (N = 7,291)** |
| --- | --- | --- | --- | --- |
| **Gi* z-score** |  |  |  |  |
| Median [Q1, Q3] | -2.57 [-2.96, -2.24] | 2.77 [2.36, 3.24] | 0.0596 [-0.851, 0.889] | 0.126 [-1.15, 1.36] |
| **Beds** |  |  |  |  |
| Median [Q1, Q3] | 79.0 [45.0, 113] | 79.0 [50.0, 113] | 80.0 [51.0, 124] | 80.0 [50.0, 120] |
| Large scale with beds ≥80 | 464 (49.9%) | 566 (49.3%) | 2687 (51.5%) | 3717 (51.0%) |
| Small scale with beds <80 | 465 (50.1%) | 583 (50.7%) | 2526 (48.5%) | 3574 (49.0%) |
| **Government-funded beds ratio** |  |  |  |  |
| Median [Q1, Q3] | 0 [0, 0.509] | 0 [0, 0.600] | 0.0556 [0, 0.615] | 0 [0, 0.600] |
| Private or partially funded with ratio <0.5 | 620 (66.7%) | 754 (65.6%) | 3286 (63.0%) | 4660 (63.9%) |
| Public or mostly funded with ratio ≥0.5 | 308 (33.2%) | 395 (34.4%) | 1892 (36.3%) | 2595 (35.6%) |
| Missing | 1 (0.1%) | 0 (0%) | 35 (0.7%) | 36 (0.5%) |
| **Staff-beds ratio** |  |  |  |  |
| Median [Q1, Q3] | 0.366 [0.275, 0.497] | 0.383 [0.266, 0.567] | 0.400 [0.286, 0.556] | 0.389 [0.279, 0.551] |
| High staffing level with ratio ≥0.4 | 366 (39.4%) | 426 (37.1%) | 2317 (44.4%) | 3109 (42.6%) |
| Low staffing level with ratio <0.4 | 477 (51.3%) | 534 (46.5%) | 2235 (42.9%) | 3246 (44.5%) |
| Missing | 86 (9.3%) | 189 (16.4%) | 661 (12.7%) | 936 (12.8%) |
| **Distance to the nearest A&E hospital^†^, *m*** |  |  |  |  |
| Median [Q1, Q3] | 1850 [1330, 2530] | 1170 [747, 1580] | 1460 [980, 2310] | 1440 [958, 2140] |
| **Outreach service of nearest hospital (x100)^†^** |  |  |  |  |
| Median [Q1, Q3] | 607 [472, 1090] | 458 [365, 475] | 459 [354, 585] | 468 [369, 591] |

**#**Spot types were derived from hotspot analyses to assess spatial clustering based on annual A&E transfer rate from RCHEs to hospitals.

†The distance to the nearest hospitals was computed using straight-line measurements. The outreach services provided by the nearest hospitals were utilized as a proxy for the outreach service levels in RCHEs.

A&E: accident and emergency; RCHEs: residential care homes for the elderly.
